# Waves of Attention to Racial Injustice on Social Media: Extrajudicial Police Killings in the United States as Focusing Events

**DOI:** 10.1177/08944393251364290

**Published:** 2025-07-31

**Authors:** Annie Waldherr, Nicola Righetti, Ryan J. Gallagher, Kira Klinger, Daniela Stoltenberg, Sagar Kumar, Dominic Ridley, Brooke Foucault Welles

**Affiliations:** 1 Department of Communication, 27258 University of Vienna, Austria; 2 Department of Communication Sciences, Humanities and International Studies, 19044 University of Urbino Carlo Bo, Italy; 3 Network Science Institute, 1848 Northeastern University, USA; 4 Department of Communication, 9185 University of Münster, Germany; 5 Institute for Media and Communication Studies, 9166 Freie Universität Berlin, Germany; 6 Department of Communication Studies, Northeastern University, USA

**Keywords:** public attention, hashtag activism, focusing events, extrajudicial killings, Black Lives Matter

## Abstract

The deaths of Black victims of police brutality, such as George Floyd, Breonna Taylor, Sandra Bland, and Philando Castile, have become focusing events and symbols for the Black Lives Matter (BLM) movement, catalyzing wide-spread public attention to racial injustice. While prior studies on hashtag activism predominantly focus on single and widely known cases, less is understood about why some incidents draw massive public attention while others do not. Addressing this gap, our study investigates the factors influencing the likelihood and size of public attention on Twitter (now X) following extrajudicial police killings. We analyzed 1.5 million tweets in response to 795 police killings between January 1, 2015, and December 8, 2016, in the United States. By examining cases on all scales, from unnoticed to prominent, we provide large-scale empirical evidence on disparities in public attention to police killings and their victims. Results indicate two distinct processes in the emergence of focusing events: While victims’ attributes such as race, age, and gender increased likelihood of receiving any attention (thresholding), variables of context and social construction were related to overall wave size (focusing).

In August 2014, the killing of Michael Brown, a Black American teenager, by a white police officer in Ferguson, Missouri sparked international outrage about police violence and racial injustice, marking what many consider to be the mainstream emergence of the Black Lives Matter (BLM) movement for racial justice (Lebron, 2017). Research has demonstrated the critical role that regular people on social media played in raising awareness about Brown’s murder and catalyzing it, along with similar atrocities, into the mainstream public sphere (Freelon et al., 2016; Jackson et al., 2020). Brown’s death, however, is not especially unusual in the United States (U.S.): between 2015 and 2019 U.S. police officers fatally shot nearly 1000 people per year, including a disproportionate number of Black Americans (Sullivan et al., 2019). These instances have been termed “extrajudicial killings” as cases where government actors kill citizens without judicial oversight or legal process (e.g., Sommer & Asal, 2019, p. 185). Most of these killings did not receive wide-spread public attention.

Yet, occasionally, Black victims of police brutality received outsized engagement on social media and beyond, such as Philando Castile, Sandra Bland, George Floyd, and Breonna Taylor. Their names and the circumstances of their deaths have become symbols and focusing events for the BLM movement and examples of successful hashtag activism in the sense of raising collective awareness for racial injustice and police violence (Jackson et al., 2020). Tagging the names of victims of police shootings soon emerged as a central practice of the BLM movement on Twitter (Wu et al., 2023), reflected also in the hashtags #SayTheirNames, #SayHerName, and #SayHisName. It has been described as “memory activism” (Ruiz, 2024, p. 257) serving not only to inform the public about ongoing police violence, but also to publicly remember victims in an online form of vigil and to connect them to prior deaths.

Most studies on hashtag activism in general, and the BLM movement in particular, however, are case studies and suffer from a positive selection bias in that they predominantly focus on instances of widely shared, trending hashtags. On the contrary, we know little about why only certain names and their corresponding hashtags receive broad social media engagement, while others do not. This is the central motivation and contribution of our study: to identify factors driving *the likelihood and size of waves of public attention on Twitter to victims of extrajudicial police killings.*

First, we situate our study at the nexus of hashtag activism, issue-attention cycles, and focusing events. We consider every extrajudicial police killing a potential *focusing event,* that is, an event centering public attention on the larger issue of social and racial injustice and potentially triggering a wave of public attention and movement mobilization. Such waves can be big or small. Therefore, we understand every amount of Twitter user engagement following the killing potential *waves*. Reviewing the state of research on hashtag activism and news sharing on social media, we extracted three groups of potentially influencing factors: (1) event-based factors relating to characteristics of the killings and the victims, (2) actor-based factors relating to the types of actors engaging in social media communication about the incident, and (3) content-based factors relating to how users post about the incident.

Second, we empirically investigated all extrajudicial police killings in the U.S. that happened between 1 January 2015 and 8 December 2016 and that were recorded in the *Fatal Force Database* curated by the *Washington Post*. We collected 1,531,919 tweets related to 795 killings and studied the influence of the factors outlined above in explaining the likelihood and size of subsequent Twitter (now X) activity. We used Generalized Additive Models (GAMs) to tackle nonlinear relationships and skewed distributions, which are common characteristics of public attention count data, particularly from networked social media.

Results indicate that there are two distinct processes in the emergence of a focusing event for a social movement: While certain attributes of the victims increase the likelihood of their names receiving any attention (*thresholding process*), a range of contextual and communicative variables comes into play for predicting overall wave size *(focusing process)*.

With our study, we contribute to a fuller understanding of the emergence of focusing events and their role for movement mobilization and subsequent waves of public attention. By systematically analyzing a very large number of extrajudicial killings and their victims, including those that went unnoticed by the broader public, we provide large-scale quantitative empirical evidence on how focusing events differ from others. These insights have implications for better understanding the mobilizing potential of extrajudicial police killings for the BLM movement. They also shed light on disparities in public attention to victims of police brutality.

## Hashtag Movements, Public Attention, and Focusing Events

Online social networks such as Twitter (now X) or Facebook offer new marketplaces of attention (Webster, 2014) where ordinary users, bloggers, media actors and algorithms constantly interact in a hybrid media system (Chadwick, 2017). This makes it easier for social movements to emerge, connect, mobilize, and gain wide-spread public attention (Bennett & Segerberg, 2012). The digital architecture of Twitter in the period of our study (2015–2016) afforded the fast formation of ad-hoc publics around hashtags (Burgess & Baym, 2020, pp. 66–67) and the emergence of waves of collective affect around key social movements (Papacharissi, 2016). Particularly for marginalized groups who are underrepresented in mainstream media and established political institutions, social media is an important means to organize support, form counter publics, and raise public awareness towards their issues and social injustices (Jackson et al., 2020).

A specific form of online protest makes use of hashtags as a means of indexing and connecting debates around issues and events: hashtag activism has been defined as a “discursive protest on social media united through a hashtagged word, phrase or sentence” (Yang, 2016, p. 13). By trending hashtags, racial injustices become widely visible and debates promoting racial justice gain momentum (Kuo, 2018). In the context of BLM, hashtags on Twitter also open digital spaces for collective memory of victims and the formation of publics countering dominant narratives (Hawkins, 2023; Ruiz, 2024).

Hashtag activism takes advantage of central mechanisms of public attention that have long been studied by communication scholars for media hypes (Vasterman, 2005) or news waves (Geiß, 2018). Public attention is characterized by steep outbursts of communication. These are triggered by certain events, called key events (Kepplinger & Habermeier, 1995), trigger events (Wien & Elmelund-Præstekær, 2009), or focusing events (Birkland, 1997), which is the term we will use in the following. Such events prompt a spiral of self-reinforcing dynamics in a way that communication leads to more communication (Vasterman, 2005; Waldherr, 2014).

In the age of social media, these basic patterns and mechanisms still apply. Particularly on Twitter, waves of engagement have been observed to be particularly steep, fast, and bursty (Lorenz-Spreen et al., 2019; Neuman et al., 2014; Oka et al., 2014; Zhang et al., 2019). They are driven by an interplay of internal network mechanisms and exogenous events (Oka et al., 2014; Stevens et al., 2018): *Internal network mechanisms* are enabled by functionalities to connect users, make visible such connections, and indicate popularity cues such as likes and shares. These processes are further fueled by algorithms that amplify popular content gaining a lot of user reactions. Particularly huge bursts of interactions on Twitter are often caused by real-life, *exogenous events* such as crises, scandals, or disasters (e.g., Lin et al., 2014; Oka et al., 2014; Pang, 2013; Stevens et al., 2018).

These real-life exogenous events serve as focusing events for public attention as they focus attention on a related, broader social issue and sustainably raise baseline attention towards it (Beckers & Van Aelst, 2019). For the hybrid media system, this phenomenon has been termed *news flashpoints* (Waisbord & Russell, 2020) and described as “attention magnets” (p. 378) pushing issues to the center of public attention across different types of media and their logics.

#BlackLivesMatter is a prime example for such a process where single focusing events led to news flashpoints increasing overall public concern about a social problem. While the hashtag was already born in 2013, the movement gained traction and crystallized around hashtags associated with specific victims of police brutality and following waves of protest and media attention (Freelon et al., 2016). Specifically, the murder of Michael Brown in 2014 sparked major protests on the streets of Ferguson, Missouri, accompanied by a digital wave of outrage on Twitter (Ince et al., 2017; Jackson & Foucault Welles, 2016). Six years later, the murder of George Floyd received more attention and amplification on Twitter than any other fatal police encounter so far. It also raised attention for subsequent, and even prior victims of police violence (Wu et al., 2023). Therefore, by generating waves of public attention, focusing events play a major role in the mobilization of movements over time (Gitomer et al., 2025).

While scholars agree that such focusing events are events attracting intensive public attention, they disagree on whether it is possible at all to identify such events a priori. Kepplinger and Habermeier (1995, pp. 372–373), for example, state that “it does not need rare, extreme and thus spectacular happenings for a key event to evolve.” Similarly, Alimi and Maney (2018, p. 762) argue “that any event could become a focusing event, as this is more a matter of social construction—how events are told, covered, or framed—than some set of intrinsic characteristics.”

Other scholars such as Birkland (1997) are unsatisfied by characterizing focusing events only post-hoc and propose that it should be possible to identify event features indicating a potential focusing event. Rare events, as well as events which cause great damage, occur suddenly, and become known to both mass audiences and political elites simultaneously, according to Birkland (1997, p. 3), have the potential to focus public attention and influence political decisions. Accordingly, Wien and Elmelund-Præstekær (2009) suggest that events suiting journalistic selection criteria of newsworthiness, violating social norms and values, sparking public debate, and allowing multiple journalistic frames and viewpoints are more likely to prompt news waves.

With our study, we contribute to solving this puzzle of what makes an event a focusing event for hashtag activism and movement mobilization. We consider every extrajudicial police killing a potential focusing event and ask which factors make it more likely to unleash this potential.

## Drivers of Collective Attention on Social Media

In the following section, we draw on two strands of literature, hashtag activism and news sharing on social media, to identify potential factors driving collective attention on social media towards victims of police brutality. We group them into event-based, actor-based, and content-based factors.

### Event-Based Factors

The idea that there are certain characteristics of an event determining how much public attention it will draw is long-standing and has been studied under the heading of news values, news factors, or newsworthiness (Eilders, 2006; Harcup & O'Neill, 2017). More recent studies found that classic news values such as conflict or proximity are also relevant for sharing news on social media (Araujo & van der Meer, 2020; García-Perdamo et al., 2018; Trilling et al., 2017), giving rise to new terms such as shareability (Harcup & O'Neill, 2017), or shareworthiness (Trilling et al., 2017).

Rudat et al. (2014) derived news factors driving the “informational value” of tweets which are relevance, unexpectedness, controversy, and negativity. These indeed were among the factors most often found to be predictive of news sharing on Twitter. A range of studies reported that posts of broad social relevance (Keib et al., 2018; Naveed et al., 2011; Valenzuela et al., 2017) or social impact (Araujo & van der Meer, 2020) were shared more often. Likewise, the news factor of deviance from expectations was supported across studies (Lamberson & Soroka, 2018; Valenzuela et al., 2017). For example, unexpected events yielded most user reactions on Facebook in a study by Salgado and Bobba (2019).

Evidence regarding the news values of controversy and negativity is less clear. Some studies reported conflict/controversy as a significant and strong predictor of news sharing on Twitter (García-Perdamo et al., 2018; Kalsnes & Larsson, 2018; Trilling et al., 2017). However, Valenzuela et al. (2017) found conflict predicted reading news posts on social media, but not sharing, a finding which Bright (2016) reported also for negative news. van der Meer and Brosius (2024) could not confirm any negativity bias with their study. Instead, Valenzuela et al. (2017) found positive news to be shared more widely, while Naveed et al. (2011) observed bad news to be retweeted more often. Yet, other scholars discovered that any kind of emotional arousal, be it negative or positive, provokes sharing (Berger & Milkman, 2012; Trilling et al., 2017).

Focusing back on our case of victims of police violence, many of the mentioned news values are present: negativity, conflict, as well as potential emotional arousal. Others, such as social relevance and social impact, we consider a matter of framing and will discuss them below. What may differ and shape public reactions are specific characteristics of the victim and the killing incident. A study by Zhang et al. (2019) examining the social media response to 59 mass shootings in the U.S. between 2012 and 2014 leads us to expect that Twitter activity might vary by demographic and other personal attributes of the victims. White et al. (2021) found that Black and Hispanic homicide victims get less news coverage, while being White, female and young was associated with more coverage. Specific personal attributes or behaviors such as wearing a weapon or acting confrontationally might align with negative stereotypes and resonate with tendencies in public discourse to delegitimize victims of police violence (Masullo et al., 2024). As, to date, there is no sufficient empirical evidence to substantiate specific hypotheses, we pose an open research question:

RQ: How are personal attributes of the victims of police violence related to the likelihood of public attention on Twitter?

Furthermore, some contextual factors of the fatal encounters, such as *place* and *time,* also vary considerably and might be influential. Geographical proximity is considered a classic factor of newsworthiness which also predicted user engagement on Twitter and Facebook (Araujo & van der Meer, 2020; Salgado & Bobba, 2019; Trilling et al., 2017; Trilling & Knudsen, 2023). While not much attention has been paid to location in the literature on online activism, specifically, general findings on the geography of Twitter usership suggest that it is likely influential. Importantly, Twitter users are clustered in large metropolitan areas (Takhteyev et al., 2012; Waldherr et al., 2024), while rural areas are underrepresented. Combined with the fact that trends on Twitter tend to emerge locally and that nation-wide trending topics in the U.S. tend to emerge from metropolitan hubs (Ferrara et al., 2013), we hypothesize that community size has a positive impact on the size of the wave of attention in activist communication.

H1 The higher the population of the community is where the killing happened, the bigger the size of the wave.

There are also indications that the time of posting a tweet constitutes a relevant predictor. In a study of retweeting patterns of activist messages, Potts et al. (2014) found that the most retweeted day of the week was Friday, followed distantly by Wednesday. Estrella-Ramón et al. (2024) found that tweets on weekdays generated more engagement. In both studies, tweets sent on weekends yielded the least resonance. Based on these findings, we hypothesize that:

H2 Killings that were first tweeted about on Fridays show bigger wave sizes than killings first tweeted on other days of the week.

### Actor-Based Factors

The formation of a wave requires actors paying attention and speaking about the event. According to hashtag activism literature, hubs in mobilizing Twitter networks often include a mix of activist organizations, citizens, and media accounts; and attention tends to focus on a few sources (e.g., Jackson & Foucault Welles, 2016; Lin et al., 2014; Papacharissi & de Fatima Oliveira, 2012). Many studies stress the importance of elite users who have either gained their status on Twitter by their number of followers, have celebrity status as political or entertainment personalities, or represent established media organizations (Carew, 2014; LeFebvre & Armstrong, 2018; Shahin et al., 2024; Wu et al., 2011).

These findings were also corroborated by a range of studies on trending topics and tweet diffusion. Asur et al. (2011) studied trending topics on Twitter and found a high number of actors involved in their propagation through the network, but they particularly stressed the relevance of traditional media outlets. In a study on user engagement with organization-related topics on Twitter, Araujo and van der Meer (2020) found that influential users (in this case politicians and political parties) as well as news media accounts drove higher user engagement. Similarly, Stevens et al. (2018) found news media accounts to drive social media activity in reaction to food scandals.

Besides media elites, so-called crowd-sourced elites were found to be relevant for the rise of social movement hashtags in networked publics (Gallagher et al., 2021; Papacharissi & de Fatima Oliveira, 2012). These are opinion leaders who are granted elite status by the crowd of social media users through retweets and mentions, but also through explicitly encouraging others to follow specific accounts of bloggers, activists, or ordinary citizens.

Both media elites and crowd-sourced elites can reach large numbers of followers in only one step of information propagation. They enable the broadcasting of news on Twitter, which Goel et al. (2016) identified as an important mechanism of tweet diffusion in a large-scale study of diffusion events (see also Umansky, 2023). As Twitter (now X) is a real-time medium, encouraging fast, but short-lived attention, the fate of tweets is decided rather quickly. For example, Bright (2016) showed that 50% of retweets of a given tweet happened within the first hour, which supports findings from an earlier, large-scale study by Kwak et al. (2010). Thus, we assume that retweets from accounts serving as hubs and reaching a larger audience in the network are particularly important in early stages of an issue-attention cycle on Twitter. We hypothesize:

H3 The earlier elite users or mass media tweet about the killing, the bigger the size of the wave.

### Content-Based Factors

The last group of factors refers to formal features of social media posts which have been found to be related to wide-spread sharing. First, evidence points towards a substantial role of hashtag use in explaining how much attention posts receive (Estrella-Ramón et al., 2024). Social movement actors reuse the same hashtags to communicatively tie together separate events. In this function, the use of hashtags is a powerful means of networked framing (Meraz & Papacharissi, 2013; Stevens et al., 2018) and serves to “create a community of like-minded people who can sustain a conversation and even mobilize offline” (Ince et al., 2017, p. 1818). Regarding police brutality in the U.S., #BlackLivesMatter is the most prominent hashtag. Others include #HandsUpDontShoot, #NoJusticeNoPeace, #IfTheyGunnedMeDown, and #Justice4All (Freelon et al., 2016).

Focusing on the Twitter discourse after the killing of Mike Brown, Blevins et al. (2019) found that hashtags of places (e.g., #Ferguson) and of proper names (e.g., #MikeBrown) were the first hashtags to go viral. Later, more ideological and conceptual hashtags such as #BlackLivesMatter or #HandsUpDontShoot became more prevalent. In a similar study, Ince et al. (2017) showed how users interacted with the BLM movement and modified its framing using hashtags over the course of the year 2014. Overall, main uses of hashtags were to express solidarity, discuss police violence, and to call for action. While hashtags focusing on the grievance of police violence first dominated, later the discussion of movement tactics became more relevant.

We hypothesize that the use of hashtags positively influences the size of waves of attention because they connect single instances of killings to the broader issue of racial injustice or to similar incidents and make them visible to the larger movement community. General evidence from tweet diffusion studies supports this assumption. For example, Jenders et al. (2013) found that tweets containing a moderate number of one to three hashtags were more likely to be retweeted than tweets without hashtags, whereas for higher numbers of hashtags the expected number of retweets decreased. Other research found that hashtag use predicted retweet frequency in general (Naveed et al., 2011; Suh et al., 2010), while Burnap et al. (2014) found that hashtag use decreased wave size and duration. On balance and considering the structural role of hashtags in connecting narratives and communities, we expect a positive influence of hashtag use on the size of the wave of attention on Twitter.

H4 The more hashtags tweets include on average, the bigger the size of the wave.

Finally, visual elements such as images, memes, and videos are also used by counterpublics to spread messages online as they attract a high degree of attention and provoke affective reactions (Leach et al., 2006). For instance, Freelon et al. (2016) showed that the most shared images of the BLM movement on Twitter were pictures of the victims, showing them happy with family and friends or showing their bodies shortly after being killed. More generally, studies found tweets containing images (or links to images), to be more likely to be retweeted (Bright, 2016; Estrella-Ramón et al., 2024; Goel et al., 2016).

H5 The more visual elements tweets include on average, the bigger the size of the wave.

## Data and Methods

We started with the *Fatal Force Database* curated by the *Washington Post*, a comprehensive record of extrajudicial police killings in the United States since 1 January 2015 (Washington Post, 2022). This database is manually collected and verified, using various sources such as local news reports, social media and public records, and detailed on the *Washington Post’s* public GitHub repository at https://github.com/washingtonpost/data-police-shootings. In the absence of comprehensive government data, scholars frequently utilize this and other open-source databases. The *Fatal Force Database* is noted for its reliability and accuracy (Feldman et al., 2017). It is considered the most complete (Peeples, 2019) and has the most restrictive inclusion criteria among its peers, demonstrating high consistency, particularly for the period analyzed in this study (Comer & Ingram, 2023). It should be noted, however, that the database only includes records of individuals in the U.S. that were killed by lethal use of a firearm.

For our analyses, we focused on the data including 1952 killings between 1 January 2015 and 8 December 2016, a time when the BLM movement was still in its earlier and formative stages. The *Fatal Force Database* includes the names of the victims along with several *personal attributes*: age, gender, race, whether they were armed and with what weapon, whether they had any signs of mental illness, whether they attacked the police (threat level), as well as whether they were fleeing and how. Additionally, the dataset comprises further *event-related attributes*: the weekday and location of the killing, the manner of death, and whether an officer was wearing a body camera.

We aggregated variables with many subcategories as follows: we grouped the victims’ age into age groups, merged marginal race categories in the data (Asian, Native American, Unknown, Other) into the category “other,”^1^ and grouped over sixty types of weapons mentioned in the dataset into five categories: bladed/blunt weapons, guns, toy weapons (replicas), unarmed/undetermined, and vehicle/other.

Two variables were added to the data: From the date of the killing, we inferred the day of the week (Monday through Sunday), and matching the location and geographic coordinates with U.S. census data, we calculated population size. For this procedure, we used the R library *tidycensus* (Walker & Herman, 2021) and Google’s Geocoding API. We identified the population size of 1904 out of 1952 events’ locations, comprising incorporated communities with legal status such as “city,” “town,” “village,” as well as unincorporated “census-designated places” (CDP) with population concentration.

Out of 1952 extrajudicial police killings, 1798 cases were complete records including all variables. These served to address our RQ on the influence of victims’ personal attributes on the likelihood of public attention on Twitter. We constructed a logistic regression model to determine the likelihood of an event being tweeted about (versus not tweeted about) in relation to the personal attribute variables of the victims as listed above and added the event-related variables including population size as controls.

To test our hypotheses, we compiled another dataset including only cases that were hashtagged and tweeted about. For each killing between 1 January 2015 and 8 December 2016, during which time we had access to Twitter’s 10% Gardenhose sample, we used the victims’ names to create hashtags like those used by BLM activists in the wake of such killings. Using various combinations of full names, nicknames, and the prefixes “justicefor” and “rip,” we created several hashtags for each victim, for example, #MichaelBrown, #MikeBrown, #RIPMichaelBrown, #JusticeForMikeBrown. In the data base of our Gardenhose sample, we identified tweets (including retweets) matching name hashtags within two weeks of the killing. Out of the 1952 killings within our data collection period, we found at least one tweet for 795. Using the tweet IDs and the Twitter API in March 2019, we rehydrated a total of 1,531,919 tweets and computed the following measures from their metadata:

We classified the tweet authors into elite versus non-elite accounts. As elite accounts we defined (a) 82 major traditional news media listed by Copeland et al. (2016), (b) accounts by key BLM activists,^2^ and all additional users ranking in the top 5% (at least 4400 followers) by follower count.^3^ Any other user was considered non-elite.

For each event, we calculated metrics for tweets, including the average number of hashtags (additional to the original hashtag used for sampling) and the mean number of visual elements (videos and pictures). We also created a variable that ranges from 1, representing the earliest hour of data collection, to 336, the final hour of data collection (which corresponds to two weeks from the date of the event) measuring when an elite user or major news media first tweeted an event-related hashtag within the two-week data collection period. The summary statistics of all metric and categorical variables are presented in the Supplementary Materials accompanying this article (see link to OSF repository below).

We operationalized our dependent variable (wave size) as the total number of tweets related to an event and applied a Generalized Additive Model (GAM) using the R package *mgcv* (Wood & Wood, 2015). We selected this analytical approach because the distribution of tweets by event was highly skewed and dispersed, and the preliminary exploratory analysis revealed complex nonlinear correlations between most variables, which violated the assumptions of linear models. In comparison to linear models, GAMs allow for covariates and responses not necessarily to be linearly correlated, and they represent a balance between the simplicity of linear models and the complexity of machine learning algorithms. GAMs are composed of smooth functions, which summarize the trend of a response measurement as a function of one or more predictors without assuming a rigid form for the dependence between variables (Hastie & Tibshirani, 1990, p. 9). Since the dependent variable consists of count data with a highly skewed and overdispersed distribution, we relied on a negative binomial model.

The variable measuring the elites’ first tweet time includes several missing values, because only a portion of the events received at least one tweet from the elites. Therefore, we fitted two models, one with and one without the variable, to ascertain the consistency of the findings independently from the impact of the missing values and the smaller sample size and corresponding statistical power. We fitted models using smooth functions of different complexity and wiggliness and tested the model assumptions, such as multicollinearity and the appropriateness of the smooth functions, using the functions provided by the *mgcv* R package and the default parameters, to represent key relationships and prevent overfitting.^4^

The data and code to reproduce the analyses and the charts in this paper, along with supplementary methodological materials and explanations, are available in the following OSF repository: https://doi.org/10.17605/OSF.IO/BSTH6.

## Results

**Figure 1.**
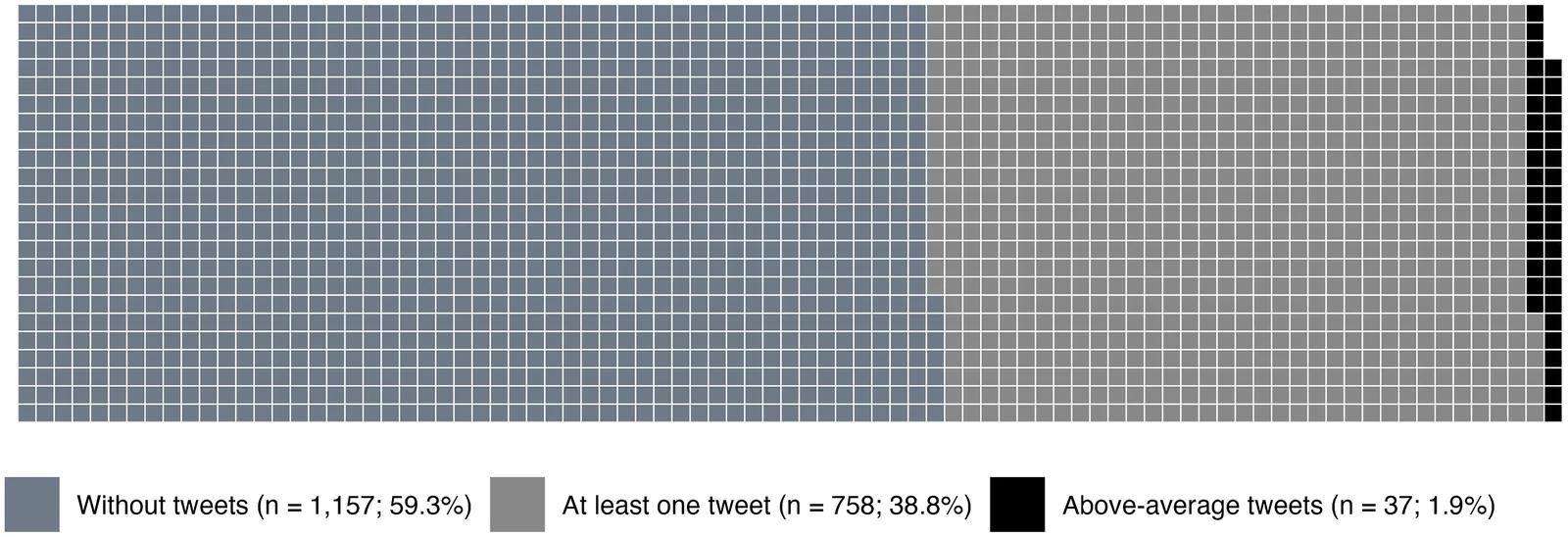
Data on 1952 fatal encounters from the Fatal Force Database curated by the Washington Post. Distribution of events in terms of Twitter coverage: events without tweets in 10% Twitter Gardenhose sample, events that received at least one tweet, and events that received above-average coverage in 2015 and 2016.

Our analysis indicates that most victims of extrajudicial police killings in our study period (59.3%) did not get any recognition in our Twitter data, while only a small portion (1.9%) received higher than average user activity related to our data, with the remaining victims obtaining a low or medium level of attention (Figure 1).

**Figure 2.**
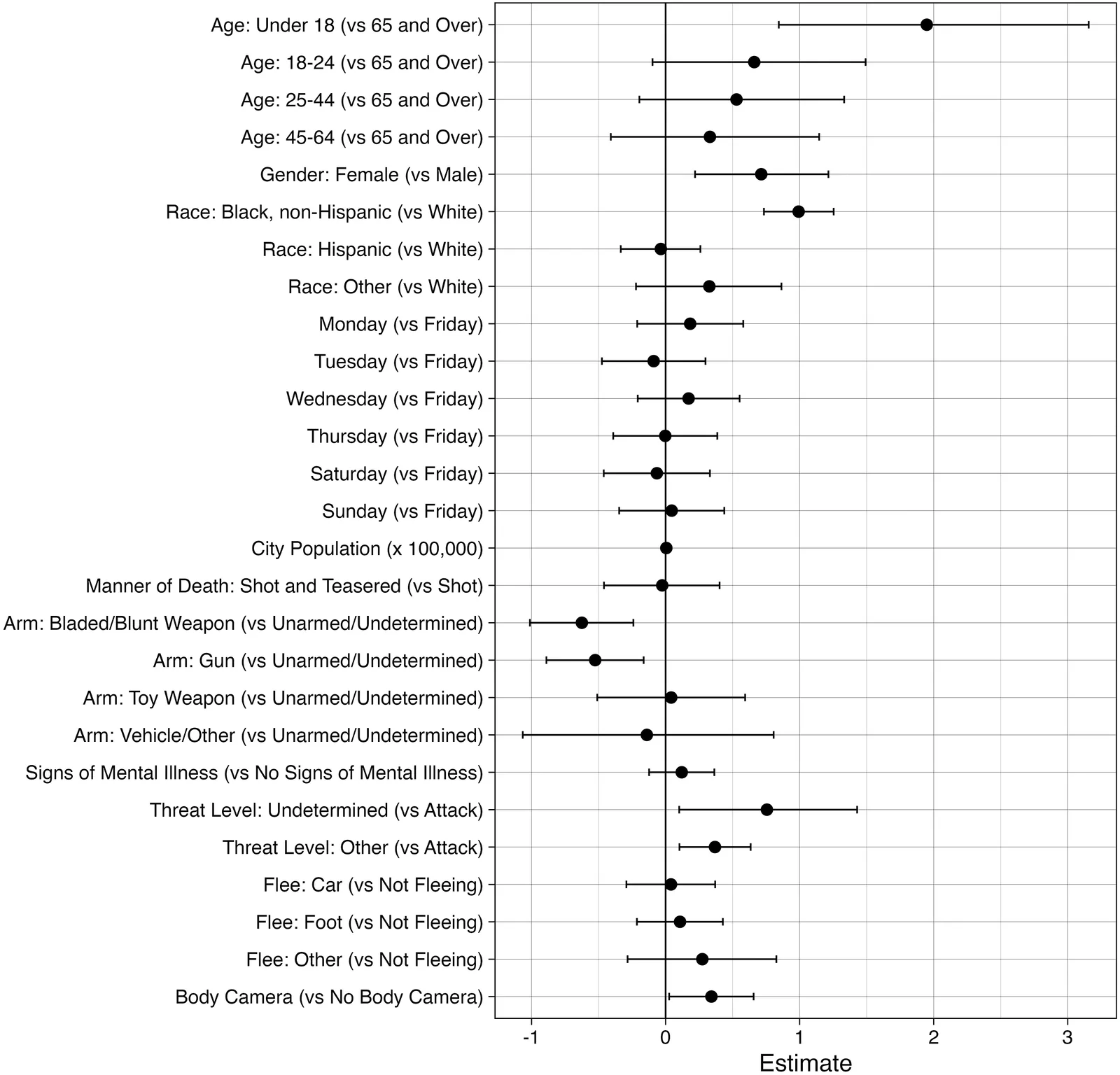
Logistic regression estimates (log odds) for likelihood of a victim’s name to be tweeted.

To answer our RQ, we examined the potential factors influencing the probability of a killing receiving attention on Twitter (Figure 2). Results show the relevance of several personal attributes for predicting the likelihood of a victim’s name being tweeted. We found that very young victims (under 18 years old) are far more likely to receive attention on Twitter compared to older individuals (65 and over). More specifically, after accounting for other predictors in the model, we found that the log odds of being tweeted are about 1.95 points higher (*p* < .01) for younger victims, indicating that they have about 7.02 times the odds of being tweeted about, compared to older victims. Female victims are also more likely to appear on Twitter than male victims (about 0.71 higher log odds than males, or 2.04 times the odds of male victims, *p* < .01). Black victims are more likely to receive attention on Twitter than white victims (about 0.99 log odds higher, or 2.70 times the odds of white victims, *p* < .001).

Furthermore, victims are more likely to be mentioned in tweets if there is no evidence of them having attacked the police prior to the killing (Threat Level: Undetermined), or if the level of the attack is classified as “other,” compared to cases of the victim attacking the police before the killing, with respective log odds of 0.76 (*p* < .05) and 0.37 (*p* < .01), or odds ratios of 2.13 and 1.45 times if the victim was alleged to have attacked the police prior to the killing. Victims who were armed with a gun, or a bladed or blunt weapon are tweeted less than victims reported to be unarmed or whose armed status is unclear. The log odds for the former are −0.53 (*p* < .01), and for the latter −0.62 (*p* < .01), indicating 0.59 and 0.54 times the odds of a victim being reported as unarmed or when their status is defined as indeterminate, respectively. The presence of a body cam also is associated (*p* < .05) with a higher likelihood of an event being tweeted (log odds increase by 0.34, corresponding to 1.41 times the odds of an event where the body cam is not present).

**Figure 3.**
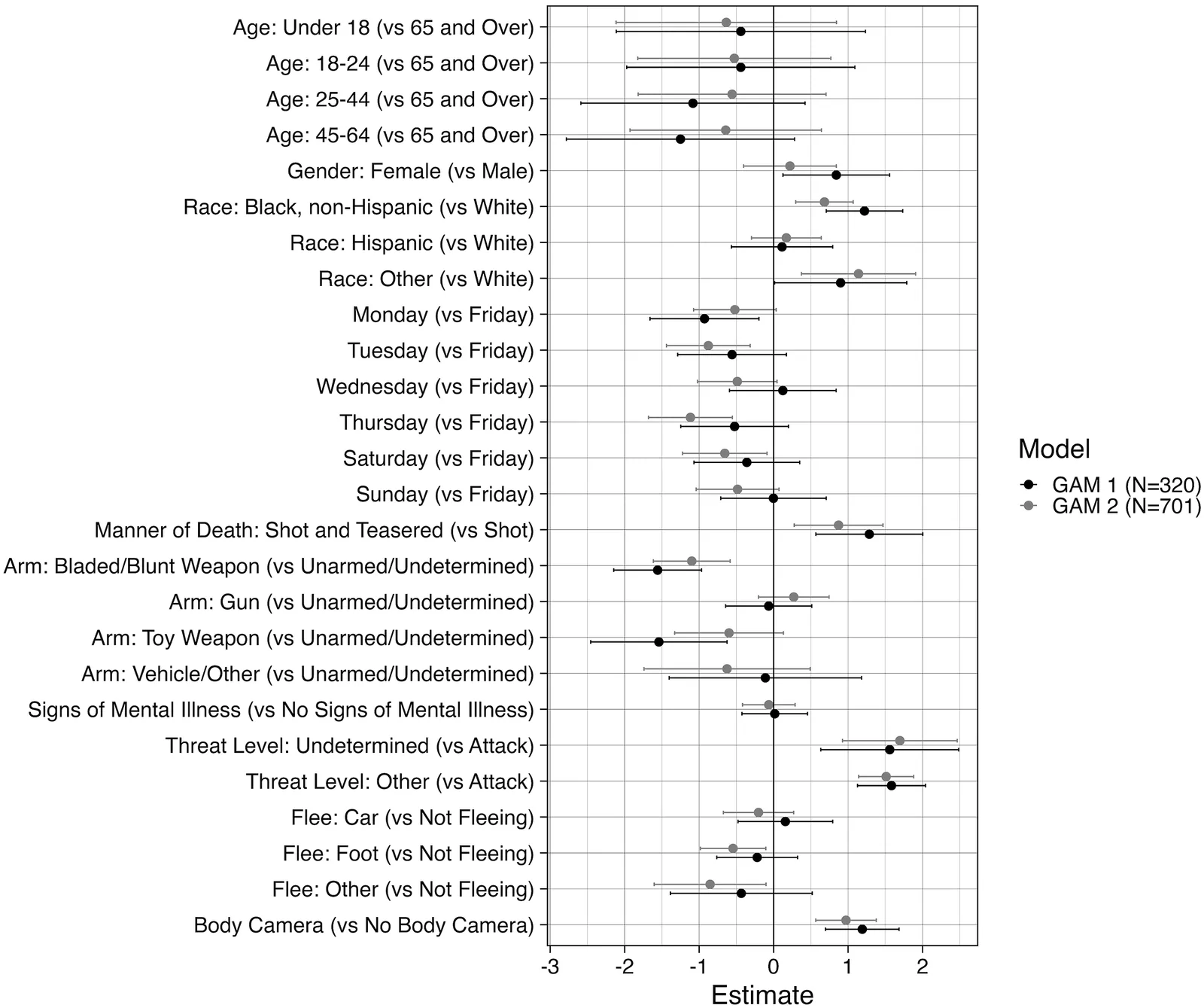
Parametric coefficients of the GAM models for the categorical variables.

**Figure 4.**
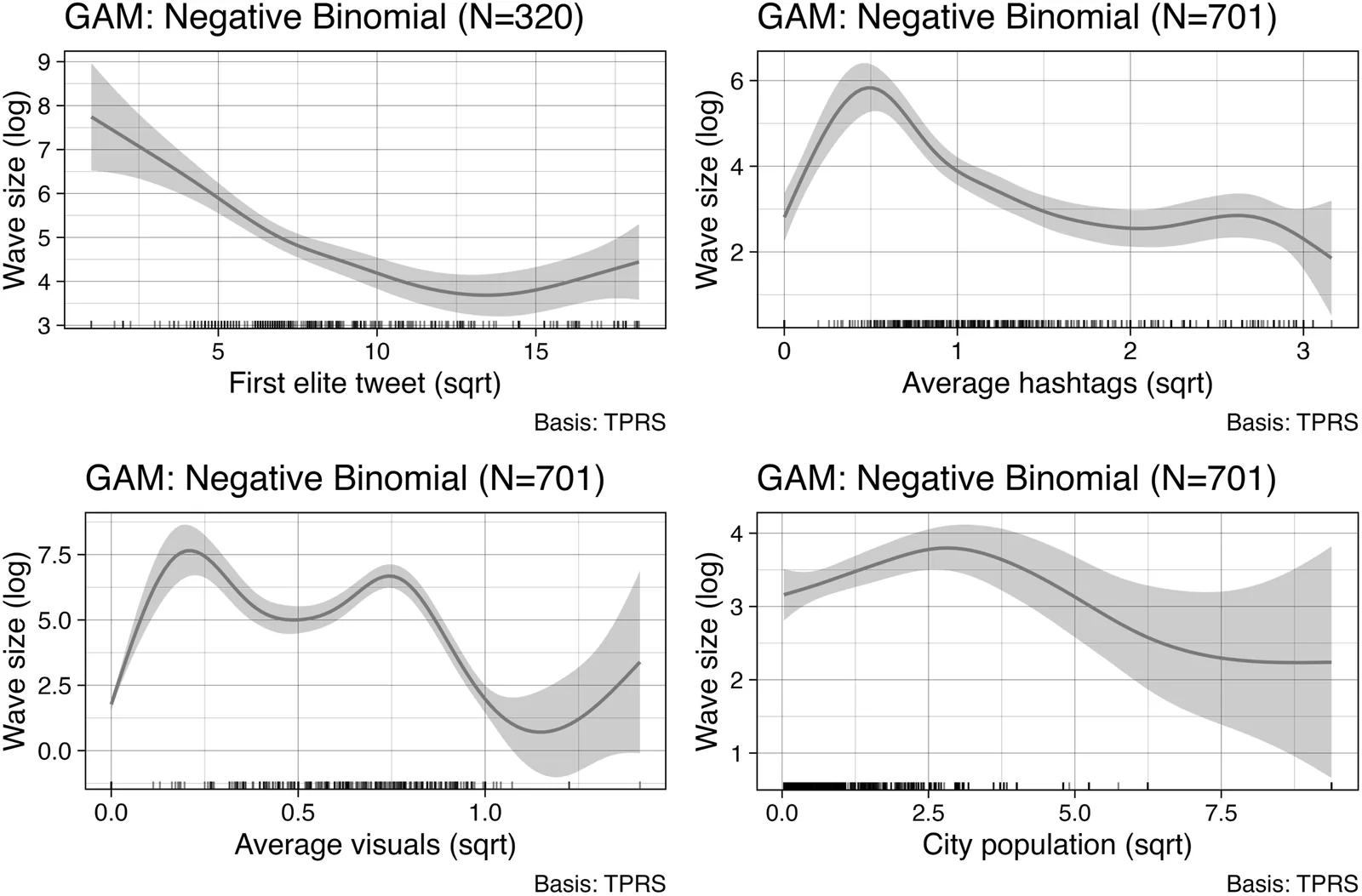
Partial effects of the nonlinear terms in the GAM models.

We then examined the hypotheses regarding the factors that might affect the size of a wave of Twitter attention using Generalized Additive Models (GAM). Figure 3 displays the relationships between the categorical predictors and the dependent variable (wave size) for two models. The first model (GAM 1) is a negative binomial model that includes the elite first tweet variable and therefore has a smaller sample size due to cases without elites’ tweets (*N* = 320). The second model (GAM 2) does not include this variable, resulting in a larger sample size (*N* = 701). Figure 4 displays the nonlinear partial effects of the metric predictors on the dependent variable, expressed on the log scale. Tables reporting the exact coefficients for both models are available in the Supplementary Materials in the OSF repository.

H1 stated that the higher the population of the community where the killing happened, the bigger the size of the wave. The findings indicate that the average number of tweets increases as population size increases, reaching a peak around places with a population of about 1 million people (Figure 4). After the peak, the average number of tweets gradually decreases for places with larger population sizes. It is worth noting that such places are rarer in the U.S. and the dataset (as shown by the rug plot on the x-axis) and the margins of errors in the model become correspondingly larger. Thus, the data only partially support H1.

H2 is also partially supported. In GAM 2 with the larger sample size, killings that were first tweeted about on Fridays are significantly related to larger wave sizes compared to Tuesday, Thursday, and Saturday. In GAM 1 based on the smaller sample, only the difference to Mondays is statistically significant (Figure 3).

The data fully corroborate H3. The earlier elite users or mass media tweet about the killing, the bigger the wave size. The findings show that the average number of tweets is higher when such users tweet about the killing early on and decreases as their response becomes less prompt (see Figure 4).

H4 proposed that the more hashtags tweets include on average, the bigger the size of the wave. Here, the models reveal a more complex, nonlinear relationship, thus not supporting H4. The average wave size increases rapidly with the average number of hashtags, reaching a peak for waves with approximately 0.5 hashtags per tweet (excluding the sampling hashtags), considering the model with the larger sample size. However, this positive relationship diminishes as the number of hashtags increases further.

Regarding H5 (The more visual elements tweets include on average, the bigger the size of the wave), the models suggest that the more visual elements are included on average in tweets concerning a given event, the greater the average volume of tweets for that event. This increase in wave size is particularly pronounced when the overall use of visual elements across the entire set of tweets related to a given event is relatively low, peaking at an average incorporation rate of about 0.05 visual elements per tweet. It then decreases and rises again, reaching a second, lower peak at about 0.55 visual elements per tweet. As the average number of visual elements increases further, up to about one visual element per tweet, the average size of the waves keeps decreasing. After that point, the events become rare, and the confidence intervals of the model correspondingly wider. The resulting pattern is not clear and hard to interpret. Therefore, our data do not support H5.

## Discussion

Our study sheds light on the driving forces of public attention towards the victims of extrajudicial police killings on Twitter (now X). Thereby, more generally, it contributes to the study of focusing events and subsequent public attention towards social injustice on social media. Combining the *Fatal Force Database* with our Twitter sample, we address an often-criticized drawback of empirical research on hashtag activism and public issue attention which is often biased towards events that already receive substantial public attention. The *Fatal Force Database*, however, includes many cases of extrajudicial police killings that had no mentions in our Twitter sample. This way, we could systematically study which victim and event characteristics are associated with the likelihood of receiving attention, and which additional factors drive the size of waves on Twitter.

Our results suggest an interplay of event characteristics and social construction. All three variable groups (i.e., event-based, actor-based, and content-based factors) proved relevant, but their differing influence in the two parts of our study leads us to conceptually differentiate between *two different processes* in the emergence of focusing events:

The first process relates to the question of which victims receive hashtags and a basic amount of attention on Twitter at all. We consider this a process of *thresholding* of potential focusing events. We found that certain characteristics of the victims made them more likely to receive attention on Twitter, namely, if they were young, female, unarmed, and did not pose a clear threat to the police or attack them. This largely corresponds to the expectations drawn from earlier research (Masullo et al., 2024; White et al., 2021; Zhang et al., 2019). However, in our case, Black victims instead of white victims were more likely to receive public attention, which may show the effectiveness of the BLM hashtag activism.

The second process relates to the size of the wave of Twitter attention. Its basic mechanism is the *focusing* of public attention as is demonstrated in the skewed distribution of our dependent variable wave size: only few victims received most attention. Compared to the thresholding process, several victim attributes lost influence in the focusing process. While being Black and not posing a clear threat to the police was also associated with higher wave size, age and gender were not significant across models; and only carrying bladed/blunt weapons, but not guns, showed a significant effect in the GAMs. Instead, contextual attributes such as timing and location proved significant, as well as early elite engagement and content-based factors such as the use of hashtags and visuals, highlighting the relevance of social construction in this stage. The differing influence of gender is particularly interesting: while women were more likely to receive hashtags on Twitter, the associated waves were smaller in size, supporting the popular perception that female victims of police violence overall receive less attention which was taken up by the movement #SayHerName (Williams, 2016).

These results have theoretical implications for research on hashtag activism: first, they show that counter publics and hashtag movements are also subject to fundamental mechanisms of the public attention economy as user engagement focuses on only few killings and their victims. Such focusing proved necessary for the successful mobilization of BLM and other movements on social media (Gitomer et al., 2025; Wu et al., 2023). However, as shown by our research, potential focusing events do not automatically trigger mobilization, but depend on being taken up and pushed by activists who connect the event to previous events through hashtags, consider timing and involve elite actors early on.

Second, BLM hashtag activism successfully counters some of the dominant attention patterns while reproducing others. For example, Black victims were more likely to receive hashtags and triggered larger waves of engagement in our data, illustrating the influence of BLM memory activism. At the same time, female, younger and unthreatening victims, that is, those deviating from stereotypical expectations, were more likely to receive engagement. Thus, hashtag activism not only counters, but also reflects dominant social narratives and inequalities.

Our analyses revealed several *nonlinear relationships* which we detected using the GAM technique. We found an inverted U-shape for population size and use of hashtags, with a positive relationship turning negative for higher values of the independent variables. This points to a *Goldilocks principle* (“just the right amount”) with more than a few hashtags being counterproductive to further attention, which has also been observed by Jenders et al. (2013). For the use of visuals, we found an even more complex pattern with several peaks which are hard to interpret and might be due to overfitting the model to the data. Testing these relationships on additional data in future studies is needed to further substantiate our findings. As nonlinearity is a core feature of public attention (Waldherr, 2014), we encourage future research to use adequate techniques to model nonlinear relationships (such as GAM).

The GAM approach also allowed us to deal with *skewed distributions*, a general challenge of social media data. Making visible the sparsity of data in specific variable ranges, the method helped us to take skewness into account when interpreting the findings, for example, regarding population size.

A main limitation of our study is related to our sampling strategy. First, our results are bound to the restrictions of Twitter’s 10% Gardenhose access, that is, not finding a hashtagged tweet for a victim in our data does not mean that there were no tweets at all. Still, we consider the 10% random sample of all tweets Twitter was offering at that time big enough to draw substantive conclusions on the overall likelihood of tweeting. Second, although we systematically concatenated the names of the victims, we cannot rule out completely that our search method overlooked some hashtags on Twitter that would have been relevant. Also, we did not collect tweets mentioning the names of the victims but not using hashtags. The two-week data collection time frame may also introduce some fuzziness as the names of victims are not always immediately known after the event, and some are never publicly revealed. Some may have been revealed and received Twitter attention only later. However, given the quick and short-lived nature of Twitter as a social media platform, it is rather unlikely that any of the victims received wide-spread attention after more than two weeks. Of course, we also do not know whether victims that went unnoticed on Twitter did receive significant coverage on other social media platforms or in the offline media. Also, we did not further analyze the textual contents of the tweets.

Our data gives important insights into a specific period from 2015 to 2016, during which Twitter played a central role in shaping and amplifying the BLM movement. Since then, the platform has changed ownership, followed by significant changes in moderation policies, algorithmic curation, and declining trust among users. Many activists have turned to other platforms. Future research will need to widen the scope of this research to other platforms. However, we are confident that the insights generated by our Twitter study in the earlier days of the BLM movement are generalizable to other social media platforms and other social movements, since the basic functions allowing amplification of public attention (i.e., hashtags and reshares) are also relevant on platforms such as Bluesky or Instagram.

Our findings are further limited by the cross-sectional research design of our study. The patterns we observe based on case-based aggregates might hide substantive self-reinforcing and reciprocal dynamics unfolding during a wave of attention over time. For example, the different groups of drivers (i.e., events, actors, and content), while conceptually independent, most likely influence each other reciprocally. Elite actors might drive the use of a specific hashtag, and in turn, a hashtag gaining momentum might draw participation from many non-elite accounts making use of social media affordances in a different way. In this case, the excessive use of hashtags or visuals might not have a limiting effect on engagement per se; we would simply observe that large waves of attention lead to forms of engagement not using hashtags or visuals. For the latter, a focusing process of its own might be at work, as only one image or audiovisual sequence can become iconic and evoke a lot of engagement.

Similarly, newsworthiness is not a stable concept, but prone to grow during a news wave due to self-reinforcing momentum (Kepplinger & Habermeier, 1995; Waldherr, 2014). Thus, elite actors are more likely to tweet under hashtags already gathering a lot of engagement. To disentangle such reinforcing dynamics within and between variables, future studies should make use of multivariate time series analysis such as vector autoregressive models. Such an analysis requires fine-grained longitudinal data of full-fledged, large-scale waves of attention. Given that only a fraction of our data would be suitable for such an analysis (see Figure 1), this would mean zooming in to comparably few cases or assembling a different data set by actively searching for big wave patterns.

In this study, however, our main aim and contribution was going beyond cases of successful hashtag campaigns and analyzing the full picture of extrajudicial police killings as potential focusing events for the BLM movement. Our data allowed us to show the uneven distribution and strong focusing of social media engagement towards only a few victims and to explore the influence of potential drivers of public attention on a large scale.

## Data Availability

The data and code to reproduce the analyses and the charts in this paper, along with supplementary methodological materials and explanations, are available in the following OSF repository: https://doi.org/10.17605/OSF.IO/BSTH6.

## Notes

1. The new “Other” category includes the original race categories “Asian” (*N* = 36), “Native American” (*N* = 27), “Other” (*N* = 12) and “Unknown” (*N* = 76) from the *Fatal Force Data Set*.

2. The list of BLM activist accounts is: @osopepatrisse, @opalayo, @aliciagarza, @shaunking, @deray, @MsPackyetti, @Nettaaaaaaaa, @samswey, @daBlockUp, @Moore_Darnell, @es_snipes, @rissaoftheway, @janaya_khan, @TamikaDMallory, @lsarsour, @elonjames, @marclamonthill, @TefPoe.

3. To get an estimate of the follower count of the tweet authors, we used a Twitter API call in the month of data collection (rehydration). This means that the follower count relates to the day of data collection and not to the day the tweet was posted.

4. Overall, the first model fits the data better than the second one. Despite the similar smoothing functions used in the two models and the fact that the results of the two models were substantially similar, the test of the smooth functions indicated that the functions of the second model were apparently unable to adequately capture the variability in the data, without sensible improvement even when the complexity of the nonlinear functions was significantly increased. This is not necessarily related to a problem with the smooth functions themselves, however, but could also result from omitting an important variable from the model, such as the “first elite tweet” variable. A more detailed explanation on the method can be found in the following online repository: https://osf.io/bsth6.
